# Supplementary material for: A Critical View of the Application of the APEX Software (Aqueous Photochemistry of Environmentally-Occurring Xenobiotics) to Predict Photoreaction Kinetics in Surface Freshwaters
Source: Molecules. 2019 Dec 18;25(1):9. doi: 10.3390/molecules25010009 (PMC7017383; doi:10.3390/molecules25010009)
Supplement: Supplementary file 1 [file molecules-25-00009-s001.zip › 3.2.4_gcc-4.4.0.zip!/3.2.4_gcc-4.4.0/3.2.4_gcc-4.4.0/doc/octave/html/interpreter/A-Sample-Function-Description.html]

A Sample Function Description - Untitled


Next: A Sample Command Description,
Up: Format of Descriptions


---

##### 1.3.5.1 A Sample Function Description

In a function description, the name of the function being described
appears first. It is followed on the same line by a list of parameters.
The names used for the parameters are also used in the body of the
description.

Here is a description of an imaginary function `foo`:

— Function: **foo** (x, y, ...)  
> The function `foo` subtracts x from y, then adds the
> remaining arguments to the result. If y is not supplied, then the
> number 19 is used by default.
>
> ```
>           foo (1, [3, 5], 3, 9)
>                => [ 14, 16 ]
>           foo (5)
>                => 14
> ```
>
> More generally,
>
> ```
>           foo (w, x, y, ...)
>           ==
>           x - w + y + ...
> ```

Any parameter whose name contains the name of a type (e.g.,
integer or matrix) is expected to be of that
type. Parameters named object may be of any type. Parameters
with other sorts of names (e.g., new\_file) are discussed
specifically in the description of the function. In some sections,
features common to parameters of several functions are described at the
beginning.

Functions in Octave may be defined in several different ways. The
category name for functions may include another name that indicates the
way that the function is defined. These additional tags include

Function File: The function described is defined using Octave commands stored in a text file. See Function Files. Built-in Function: The function described is written in a language like C++, C, or Fortran, and is part of the compiled Octave binary. Loadable Function: The function described is written in a language like C++, C, or Fortran. On systems that support dynamic linking of user-supplied functions, it may be automatically linked while Octave is running, but only if it is needed. See Dynamically Linked Functions. Mapping Function: The function described works element-by-element for matrix and vector arguments.
